# Accelerating Cementite Precipitation during the Non-Isothermal Process by Applying Tensile Stress in GCr15 Bearing Steel

**DOI:** 10.3390/ma11122403

**Published:** 2018-11-28

**Authors:** Feng Wang, Dong-Sheng Qian, Peng Xiao, Song Deng

**Affiliations:** 1 School of Materials Science and Engineering, Wuhan University of Technology, Wuhan 430070, China; wangfengwhut@163.com (F.W.); whutxiaopeng@163.com (P.X.); 2 Hubei Key Laboratory of Advanced Technology for Automotive Components, Wuhan 430070, China; guoheng0722@126.com

**Keywords:** GCr15 bearing steel, cementite precipitation, stress filed, kinetic analysis

## Abstract

In this work, the non-isothermal process of GCr15 bearing steel after quenching and tempering (QT) under different tensile stress (0, 20, 40 MPa) was investigated by kinetic analysis and microstructural observation. The Kissinger method and differential isoconversional method were employed to assess the kinetic parameters of the microstructural evolution during the non-isothermal process with and without applied stress. It is found that the activation energy of retained austenite decomposition slightly increases from 109.4 kJ/mol to 121.5 kJ/mol with the increase of tensile stress. However, the activation energy of cementite precipitation decreases from 179.4 kJ/mol to 94.7 kJ/mol, proving that tensile stress could reduce the energy barrier of cementite precipitation. In addition, the microstructural observation based on scanning and transmission electron microscopy (SEM and TEM) shows that more cementite has formed for the specimens with the applied tensile stress, whereas there is still a large number of ε carbides existing in the specimens without stress. The results of X-ray diffraction (XRD) also verify that carbon in martensite diffuses more and participates in the formation of cementite under the applied tensile stress, which thus are in good agreement with the kinetic analysis. The mechanisms for the differences in cementite precipitation behaviors may lie in the acceleration of carbon atoms migration and the reduction of the nucleation barrier by applying tensile stress.

## 1. Introduction

GCr15 bearing steel, with high carbon and chromium concentrations, is widely used for the rolling elements in bearing. It is routinely treated by spheroidising, quenching and tempering (QT) to obtain a microstructure comprised of spherical cementite, small amount of retained austenite and tempered martensite. Generally, bearing needs to be served under high speed and high load conditions. It has been reported [1,2] that the service temperature of ordinary bearing may reach 200 °C, while the aerospace bearing can be serviced up to 300 °C. Moreover, deterioration of bearing performance may occur during the cyclic contact loading. Microstructural development under the temperature and stress field is the crucial factor influencing the service life of the bearing steel [2,3]. Therefore, clarifying the microstructural evolution caused by the temperature and stress field to understand the mechanism of performance degradation is always an attractive topic.

Many researchers have used transmission electron microscope [4], resistivity [5], thermal analysis [6,7] and other methods to study the microstructural evolution of bearing steel during tempering or aging. There are also some studies focusing on the effect of stress on the transformation of retained austenite [8,9] and carbide precipitation [10] without an applied temperature field. Nevertheless, these studies do not consider the coupling effect of the stress field and temperature field on the microstructural transformation behaviors. Therefore, it is necessary to study the effect of stress on microstructures development during the non-isothermal process. Until now, some studies have been concentrated on the effect of stress on phase transformation behaviors, such as isothermal ferrite and pearlite transformations [11], austenite decomposition [12], martensitic phase transformations [13], bainitic phase transformation [14] and so on. However, to our best knowledge, there is still no research concerning on the microstructural transformation, and especially the cementite precipitation behaviors of GCr15 bearing steel during the non-isothermal process under applied stress.

As the non-isothermal process is close to the high-temperature tempering process for GCr15 bearing steel, the microstructure evolution during tempering should be clarified first. According to the theory of tempering, the process of tempering can be divided into several stages with the increase of temperature [15]: (1) Stage 0, 0~80 °C, the migration of carbon atoms to dislocation and defects [16]; (2) Stage I, 100~200 °C, the precipitation of ε/η transition carbides [17]; (3) Stage II, 250~350 °C, the decomposition of retained austenite [18]; (4) Stage III, 300~400 °C, the conversion of the transition carbide into cementite [19].

It is well known that the finished bearing products have gone through Stage 0 and Stage I after the traditional QT treatment, so the aim of the present work is mainly to investigate the microstructural evolution (Stage II and Stage III) during the higher tempering temperature under different tensile stresses. The kinetics parameters of microstructural development were compared and analyzed considering the effect of applied tensile stress. At the same time, the impact of applied stress on microstructural evolution during the non-isothermal process was analyzed based on microstructural analysis. In particular, the mechanism of tensile stress on cementite precipitation was discussed.

## 2. Experimental and Theory

The material used in the current study was GCr15 bearing steel, with the nominal composition as presented in Table 1. The experimental materials were austenitized at 860 °C for 15 min and followed by quenching in oil at 60 °C for 5 min. Then they were tempered at 160 °C for 2 h. After the QT heat treatment, the steel was designed to be treated under the couple action of the stress and temperature field.

In order to observe the microstructural evolution during the non-isothermal process under different tensile stresses, the specimens with a size of ϕ 10 mm × 105 mm were subjected to a continuous tensile stress (0, 20 and 40 MPa) and heated from room temperature to 300 °C (considering the limit service temperature of bearing and the temperature range of stage II and stage III) at the heating rate of 10 °C/min by means of a Gleeble 3500 thermo-mechanical simulator. The microstructure of these specimens was examined by a field-emission scanning electron microscope (FESEM, FEI Quanta 450, Hillsboro, OR, USA) and transmission electron microscopy (TEM, JEOL-2100F, Akishima, Japan). The specimens for FESEM were etched in an alcohol solution containing 4% nitric acid (volume fraction) for 10 s. The specimens for TEM were prepared by mechanically polishing and then electro-polishing in a twin-jet polisher (Struers, TenuPol-5, Ballerup, Denmark) using a solution of 10% perchloric acid and 90% acetic acid. Moreover, X-ray diffraction (XRD) data were recorded on a D/MAX-RB diffraction analyser (Rigaku, Tokyo, Japan) at 12 kW. 

Activation energy, as an important kinetic parameter, can be used to evaluate the difficulty of phase transformation. The Kissinger method [20,21] and isoconversional method [22,23] are the most widely used methods to calculate activation energy, and they are usually combined with thermal analysis. In this study, for analysing the influence of tensile stress on the kinetic of microstructural evolution during the non-isothermal process, a group of specimens was heated to 500 °C under a 40 MPa tensile stress at different heating rate of 10, 15 and 20 °C/min with the aim of calculating the activation energy. It was noted that the thermal expansion curves were recorded by measuring the variation in diameter during the non-isothermal process. Considering the activation energy determined from the thermal expansion curves and heat flow curves (both as functions of the heating rate) is really close [7,24], the kinetic parameters of microstructural evolution without stress was obtained by differential scanning calorimetry (DSC) experiments using a STA449F3 thermal analyzer (Netzsch, Selb, Germany) as a comparison. The specimens for DSC were cut into ϕ 4 mm × 0.6 mm and then heated from ambient temperature to 500 °C at different heating rates of 5, 10, 15 °C/min, respectively. Here, pure aluminum disks were used as the reference material, and the baseline was determined by performing a rerun at the same heating rate. 

The effective activation energies of different stages during the non-isothermal process were calculated by using the Kissinger equation based on the fact that the peak temperature depends on the heating rate:

(1) $$\mathrm{In}\left(T_{P}^{2}/\emptyset \right)=Q/RT_{P}+\mathrm{const}$$

where $T_{P}$ is the peak temperature, ∅ is the heating rate, and R is the gas constant (R = 8.314 kJ/mol). By plotting $\mathrm{In}\left(T_{P}^{2}/\emptyset \right)$ as a function of $1/T_{P}$, the activation energy can be obtained.

The differential isoconversional method was also employed to obtain the kinetic parameters of microstructural evolution during the non-isothermal process under different tensile stress. In this method, the reaction rate can be assumed to be a function of temperature (k(T)) and converted fraction (f(α)), which is expressed as:

(2) dα/dt=k(T)f(α)

where α and *T* is the converted fraction and temperature, respectively. k(T) can be obtained by the Arrhenius equation as:

(3) k(T)=A·exp(−Q/RT)

where A is the pre-exponential factor and Q is the activation energy. By combining Equations (2) and (3) and taking the equation to a logarithm, then the differential isoconversional method can be proposed, as follows:

(4) ln(dα/dt)=ln[A·f(α)]−Q/RT

For the non-isothermal process with a constant heating rate ∅, Equation (4) can be expressed as:

(5) ln[∅·(dα/dt)]=ln[A·f(α)]−Q/RT

By plotting ln[∅·(dα/dt)] as a function of 1/T, the slope indicates a value of Q/R. Then the curve of the activation energy varying with the converted fraction can be further obtained. 

## 3. Results

**Figure 1:**
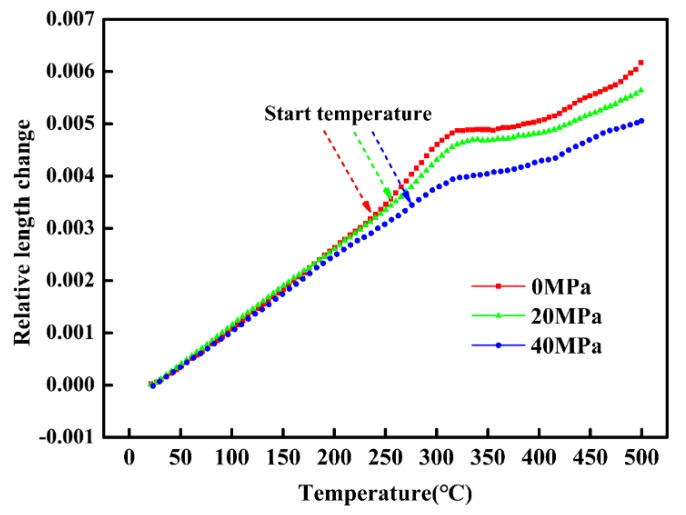
The thermal expansion curves during the non-isothermal process under different tensile stress.

The thermal expansion curves of continuous heating up to 500 °C at the heating rate of 10 °C/min under different tensile stress is shown in Figure 1. It can be found that the relative dimensional change first increases with increasing temperature linearly. When the temperature exceeds 200 °C, the slopes between temperature and relative dimensional change begins to increase and then decline. With the temperature further increasing to 400 °C, the slopes no longer change. According to the theory of tempering, the specimens may have gone through Stage 0 and Stage I, thus the slopes remained unchanged when temperature was below 200 °C. However, it is known that Stage II leads to expansion and Stage III decreases the dimension. Therefore, the variation of slopes during 200~400 °C is closely related to the microstructural evolution in Stage II and Stage III.

Based on the above thermal expansion curves (Figure 1), the starting and ending temperatures of slope change was obtained by the tangents method [25] as shown in Table 2. In fact, according to the theory of tempering, the starting temperatures of slope change can represent the beginning of retained austenite decomposition (Stage II), while the ending temperature of slope change can reflect the complete formation of cementite (Stage III). The results listed in Table 2 clearly show that the starting temperature of retained austenite decomposition increases from 238 °C to 276 °C when the applied tensile stress increases from 0 MPa to 40 MPa. This may be due to the increase of stability of retained austenite induced by applied mechanical stress. However, it can be further found that the ending temperature of cementite formation obviously decreases from 474 °C to 418 °C with the increase of tensile stress, which may indicate a premature formation of cementite. 

**Figure 2:**
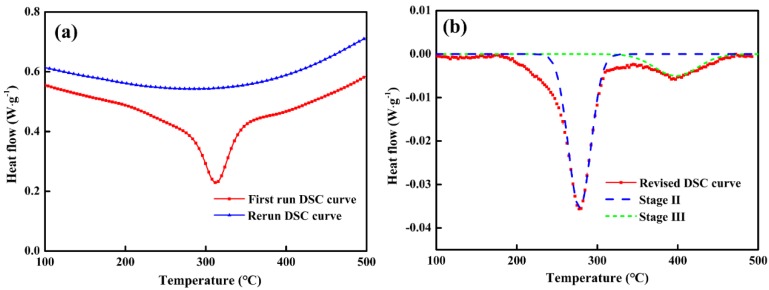
(**a**) The differential scanning calorimetry (DSC) curves at heating rate of 5 °C/min; (**b**) revised DSC curves after subtracting baseline.

Furthermore, the activation energy of microstructural evolution without stress and with 40 MPa tensile stress were obtained by DSC testing and thermo-mechanical simulator experiments, respectively. Figure 2a illustrates the representative DSC curves at heating rate of 5 °C/min, where it can be seen that the first run curve (solid line) contains a significant heat flow peak in the range of 200~400 °C. Meanwhile, the rerun curve (dash line) had no visible peaks, which was quite necessary to act as a baseline to remove the influence of experimental instruments and environment.

By subtracting the baseline and dividing by the mass of the specimens, the revised DSC curve was obtained as shown in Figure 2b. It is obvious that no heat flow peak can be observed when the temperature is less than 200 °C, indicating that QT-treated specimens have gone through Stage 0 and Stage I. In the subsequent non-isothermal process, two heat flow peaks appeared in the range of 200~260 °C and 300~400 °C, which corresponds to Stage II and Stage III, respectively. Furthermore, by fitting the revised DSC curve, the curves of retained austenite decomposition (Stage II) and carbide transformation (Stage III) were both obtained, as shown in Figure 2b.

**Figure 3:**
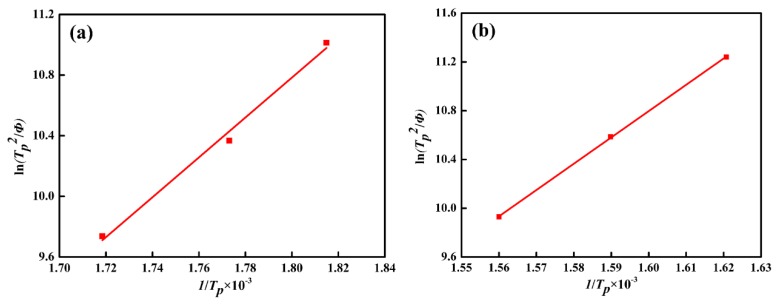
Kissinger analysis (l$n\left(T_{P}^{2}/\emptyset \right)$ versus $1/T_{P}\times 10^{-3}$ ) for the determination of the individual activation energy of Stage II (**a**) and Stage III (**b**) without applied stress.

Accordingly, the peak temperatures of Stage II and Stage III at different heating rates were listed in Table 3. As the heating rate increases, their peak temperatures gradually shift higher, indicating that higher heating rate will delay the transformation of Stage II and Stage III. According to the Kissinger method, the Kissinger straight lines of two stages can be obtained respectively, as shown in Figure 3. As a result, the activation energy of retained austenite decomposition was calculated to be 109.4 kJ/mol, and the activation energy of cementite precipitation was 179.4 kJ/mol, which is consistent with the report in literature [7].

**Figure 4:**
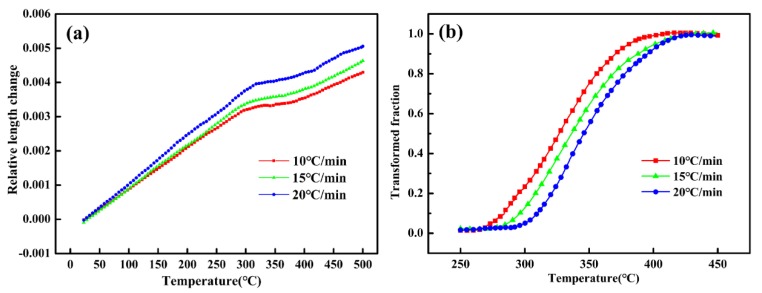
The thermal expansion curves (**a**) and transformed fraction curves (**b**) under 40 MPa tensile stress at a heating rate of 10, 15, 20 °C/min.

Figure 4a indicates the thermal expansion curves under 40 MPa tensile stress at different heating rate. As the most common method for analyzing the thermal expansion curves, the leverage theorem was used in this study and the transformed fraction curves were shown in Figure 4b. It can be observed that higher heating rate will result in an increase of the temperature of phase transformation, which is well consistent with the previous DSC results. In addition, the median temperatures (transformed fraction = 0.5) were listed in Table 4 as well, which was selected to calculate the activation energy by means of the Kissinger method. Consequently, the activation energy obtained by leverage law was 102.1 kJ/mol. However, considering that the transformed fraction curves calculated by the leverage law could not distinguish Stage II and Stage III, a follow-up calculation was thus performed using the isoconversional method.

**Figure 5:**
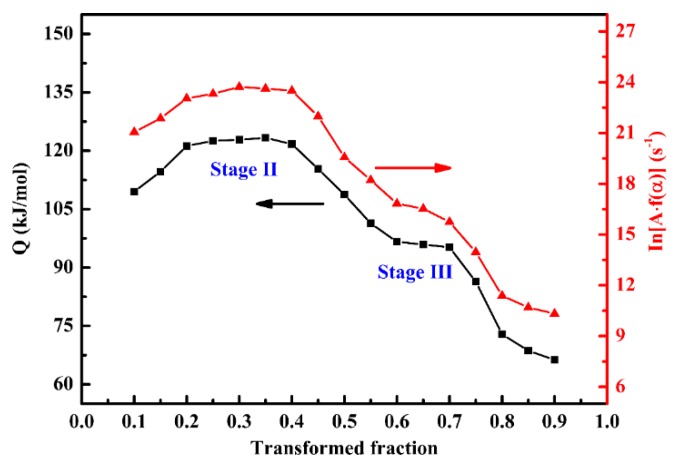
The activation energy and modified pre-exponential factor with respect to the transformed fraction.

The isoconversional method can effectively reflect the change of activation energy during the whole process, which can be considered as a more accurate supplement for the Kissinger method. A detailed activation energy and modified pre-exponential factor (ln[A·f(α)]) with respect to different transformed fraction were estimated using differential isoconversional method, as illustrated in Figure 5. It can be found that the activation energy first increases to 121.5 kJ/mol and then decreases to 72.8 kJ/mol with the increasing transformed fraction. During the early stage (0.2 ≤ α ≤ 0.4) and later stage (0.6 ≤ α ≤ 0.7), there are two platforms whose average activation energy are 121.5 kJ/mol and 94.7 kJ/mol, respectively. In fact, the platforms can be interpreted as different stages as reported by Wang et al. [26]. In the present work, according to the reaction sequence of Stage II and Stage III and kinetics analysis by the Kissinger method, the two platforms can be interpreted as the two stages (retained austenite decomposition and cementite precipitation). Meanwhile, the average value of these two stages was 108.1 kJ/mol, which was close to the activation energy obtained by Kissinger method (102.1 kJ/mol) as before. Compared with the activation energy without stress, it was found that the activation energies of Stage II and Stage III changed significantly. Under the applied tensile stress, the activation energy of the decomposition of retained austenite slightly increase to 121.5 kJ/mol, while the cementite precipitation was significantly accelerated due to the decreased activation energy of cementite precipitation (from 179.4 kJ/mol to 94.7 kJ/mol). Moreover, the results show that the variation of modified pre-exponential factor (ln[A·f(α)]) presented similar trends to those of the activation energies.

**Figure 6:**
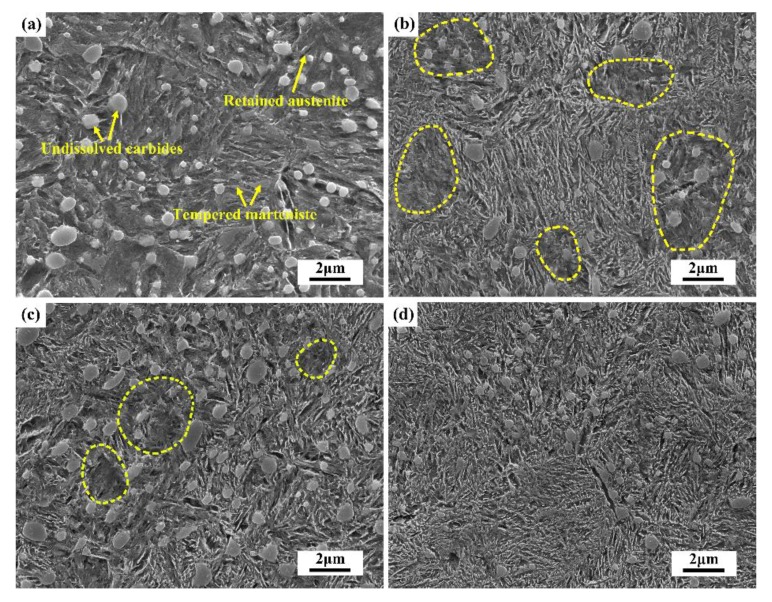
Typical field-emission scanning electron microscope (FESEM) microstructure of the (**a**) quenching and tempering (QT) treated specimens, and the QT treated specimens after heating to 300 °C under tensile stress of (**b**) 0 MPa, (**c**) 20 MPa and (**d**) 40 MPa.

To observe the microstructural evolution during the non-isothermal process, the QT treated specimens were heated to 300 °C and then air cooled to ambient temperature for microstructural analysis. Figure 6a shows the FESEM microstructure of the QT treated specimens which comprises undissolved spherical cementite, retained austenite and tempered martensite. Figure 6b–d illustrates the microstructure of the specimens after heating to 300 °C with different tensile stress, where it can be seen the matrix is mainly consisted of undissolved spherical carbides and precipitated needle-like cementite. In addition, the region highlighted by the yellow oval is poor of the precipitated cementite, indicating that there is still a large amount of cementite not formed for the specimens without tensile stress. However, more cementite has formed for the specimens with increasing tensile stress, which thus proves that the applied tensile stress can accelerate the formation of cementite efficiently.

**Figure 7:**
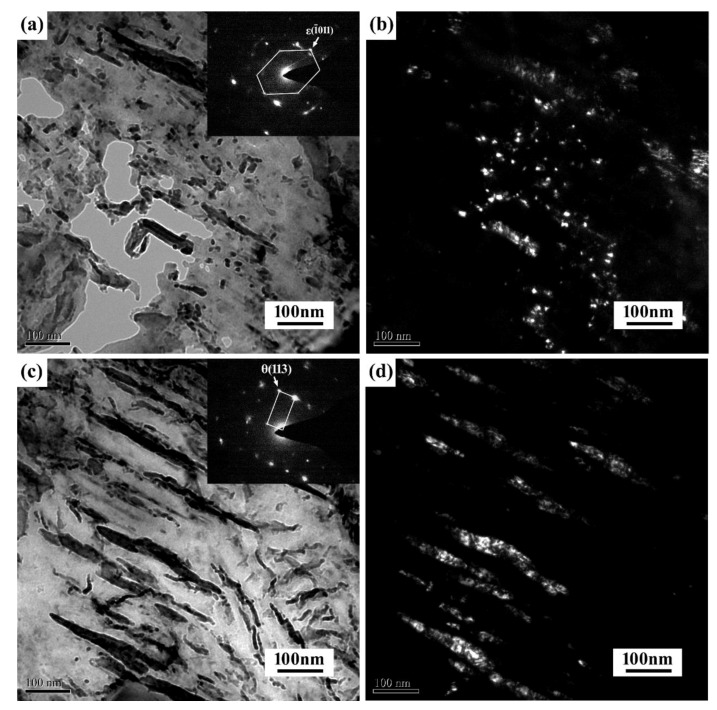
The presence of ε-carbides and θ-carbides for the specimens (**a**) without stress and (**c**) with 40 MPa, respectively. Where (**b**,**d**) are the corresponding dark field of (**a**,**c**), respectively.

Figure 7 illustrates the TEM micrographs and corresponding dark field of the microstructure of the specimen with and without 40 MPa stress, in which typical morphologies of carbides are observed. As shown in Figure 7a, some nano-size particles were found for the specimens without stress. These particles were identified using selected-area electron diffraction (SAED) to be ε-carbides. For the specimens with 40 MPa stress (Figure 7b), the needle-like precipitates presented in the matrix were identified to be θ-carbides (cementite), which is consistent with the SEM results. It can be inferred that a large number of ε-carbides still exist in the specimens without applied stress, and they have not been transformed into stable cementite at 300 °C. However, numerous stable θ-carbides (cementite) have formed for the specimens with 40 MPa stress, demonstrating again that the applied stress can accelerate the formation of cementite during the non-isothermal process.

**Figure 8:**
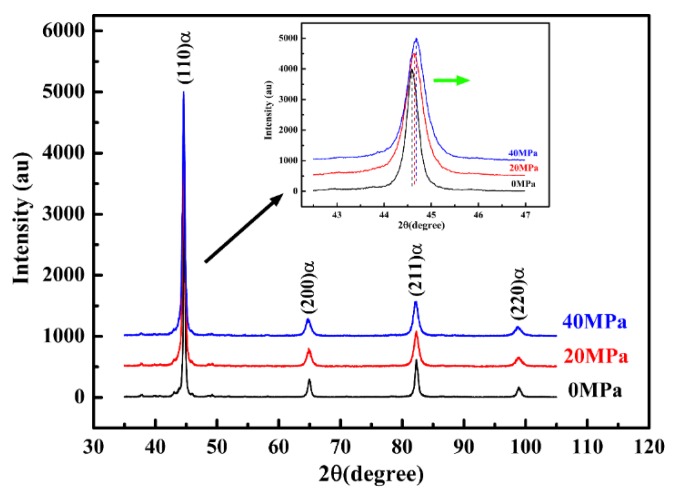
The X-ray diffraction (XRD) pattern of the specimens after being heated to 300 °C under different tensile stress.

The results of the XRD diffraction pattern (Figure 8) clearly show that the retained austenite has been completely decomposed after heating to 300 °C, regardless of the applied tensile stress. In addition, according to the observation of the (110)α diffraction peak (as seen in the insert), it can be seen that the martensite diffraction peak shifts to a higher angle with the increase of applied tensile stress. It has been reported that the diffraction peak information of martensite is closely related to the carbon content in martensite [27]. In the present work, the higher diffraction angle of (110) martensite indicates the lower carbon content of tempered martensite for the specimens subjected to tensile stress [28]. Therefore, it can be inferred that interstitial carbon atoms in martensite diffuse more and participate in the formation of cementite under the applied tensile stress.

## 4. Discussion

The mechanism of cementite formation was mainly determined by the diffusion of carbon atoms in the long range or short range. Chen [23] and Li [29] have studied the diffusion behavior of interstitial atoms in α-Fe under the strain field and they find that with the increase of tensile stress, the diffusion barrier decreases and atom diffusion gradually becomes easier. In this study, when external tensile stress is applied, the cubic crystal produces a weak elastic deformation along the tensile direction, then the diffusion barrier and atomic transition distance changes, which affects the diffusion rate of the carbon atoms. As a consequence, the applied tensile stress will exert a significant influence on the migration of carbon atoms and the formation of cementite. 

It is reported by Kim [30] that the extra lattice energy arose from the presence of defect (including dislocations, grain boundaries, twins, etc.) in the matrix could lead to a reduction of nucleation energy barrier of cementite. In the present work, according to the first law of thermodynamics, the change of internal energy of the lattice can be expressed as follows:

(6) dU=dQ−dW

where dQ and dW is the heat absorption and energy dissipation of the lattice, respectively. Assuming that the elastic strain of lattice (Δl) occurs along the tensile direction when tensile stress (*f*) is applied, the heat absorption and energy dissipation are obtained based on the second law of thermodynamics [31]:

(7) dQ=TdS

(8) dW=PdV−fΔl

where *T* and *S* is the temperature and surface area of the lattice, respectively; *P* and dV is the pressure and the volume change, respectively. By substituting Equations (7) and (8) into Equation (6), the modified internal energy change in the lattice (Equation (6)) can be expressed as:

(9) dU=TdS−PdV+fΔl

For crystals, PdV=0, So Equation (9) can be simplified as:

(10) dU=TdS+fΔl

It should be noted that for the non-isothermal process, the lattice must be in the endothermic state, so the value of heat absorption will be positive (TdS>0). Moreover, since the value of tensile stress is positive (f>0), the change of internal energy in the lattice (dU) will increase compared with those without applied tensile stress under the same temperature. Therefore, it can be inferred that the applied tensile stress favors the increase of internal energy in the lattice, thereby leading to a reduction of the nucleation of the energy barrier for the crystal core with a same size. This is also the reason for the obvious decrease in activation energy of cementite formation when the tensile stress is applied.

## 5. Conclusions

The kinetic parameters of the microstructural evolution during the non-isothermal process with and without tensile stress were investigated using Kissinger and isoconversional methods. The observation of microstructure was conducted by means of scanning and transmission electron microscopy and X-ray diffraction after heating to 300 °C. The corresponding conclusions drawn are as follows:

(1) The activation energy of retained austenite decomposition slightly increases from 109.4 kJ/mol to 121.5 kJ/mol with the increase of tensile stress, which indicates the applied tensile stress is in favor of the stabilization of retained austenite. Additionally, the applied tensile stress not only lowers the end temperature of cementite formation, but also leads to a decrease of the activation energy of cementite precipitation from 179.4 kJ/mol to 94.7 kJ/mol, thereby proving that tensile stress can reduce the energy barrier of cementite precipitation.
(2) The microstructural observation shows that more cementite has formed for the specimens with the applied tensile stress, whereas there is still a large number of ε carbides existing in the specimens without stress. The results of XRD also verified that carbon in martensite diffuses more and participates in the formation of cementite under the applied tensile stress. Therefore, these results are in good agreement with the kinetic analysis, which together proves that the applied tensile stress can accelerate the precipitation of cementite during the non-isothermal process.

## Figures and Tables

**Table 1:** The chemical composition of GCr15 bearing steel (wt %).

C	Cr	Mn	Si	P	S	Fe
0.960	1.430	0.350	0.270	0.012	0.002	Bal.

**Table 2:** The starting and ending temperatures of slope change under different tensile stress.

Tensile Stress	0 MPa	20 MPa	40 MPa
Starting temperature (Stage II)	238 °C	257 °C	276 °C
Ending temperature (Stage III)	474 °C	421 °C	418 °C

**Table 3:** The peak temperatures of Stage II and Stage III without applied stress at different heating rates.

Heating Rates	Peak Temperatures	
	Stage II	Stage III
5 °C/min	278 °C	344 °C
10 °C/min	291 °C	353 °C
15 °C/min	309 °C	368 °C

**Table 4:** The median temperature under 40 MPa at different heating rates.

Heat Rate	10 °C/min	15 °C/min	20 °C/min
Median temperature	327 °C	337 °C	346 °C
